# Actual status of early intervention services in Germany

**DOI:** 10.1192/j.eurpsy.2023.82

**Published:** 2023-07-19

**Authors:** A. Bechdolf

**Affiliations:** Department of Psychiatry, Vivantes/Charité Universitätsmedizin Berlin, Berlin, Germany

## Abstract

**Abstract:**

We will give an overview of the status of early intervention services for psychosis in Germany. We recently established a website which provides people in Germany with the nearest early detection an intervention service available (https://www.psycho-check.com). However, the overall implementation rate of early detction in Germany is quite heterogenous. We will also present recent research and ongoing projects from Germany including the first evaluation of specialized inpatient services for early psychosis, first evaluations of Individual placement and support and a mindfulness based group intervention in people with early psychosis as well as a newly desigend youth mental health service called soulspace (www.soulspace-berlin.de).

**Disclosure of Interest:**

None Declared

